# Multiple image encryption algorithm using channel randomization and multiple chaotic maps

**DOI:** 10.1038/s41598-024-79282-6

**Published:** 2024-12-23

**Authors:** Khalid M. Hosny, Yasmin M. Elnabawy, Rania A. Salama, Ahmed M. Elshewey

**Affiliations:** 1 Department of Information Technology, Faculty of Computers and Information, Zagazig University, P.O. Box 44519, Zagazig, Egypt; 2 Department of Computer Science, Faculty of Computers and Information, Suez University, P.O. Box 43221, Suez, Egypt; 3 Department of Information Technology, Faculty of Computers and Information, Suez University, P.O. Box 43221, Suez, Egypt

**Keywords:** Multiple image encryption, Color images, Channel randomization, Baker chaotic map, Henon Map, 2-D logistic chaotic map, Engineering, Mathematics and computing

## Abstract

Developing robust and secure image encryption methods for transmitting multiple images in batches over unprotected networks has become imperative. This necessity arises from the limitations of single-image encryption techniques in managing the escalating volume of extensive data. This paper introduces a novel three-layer multiple-image encryption (MIE) technique to encrypt batch images based on three 2D-chaotic maps. All multiple-color images are divided into RGB channels in the first layer. The images in each channel go through a randomization process to be arranged at random before being combined to form a single image (batch) used as the input for the following layer. In the second layer, chaotic sequences for scrambling pixels in each channel independently are generated using Baker, Henon, and 2-D Logistic chaotic maps, resulting in a scrambled image. The diffusion process is applied in the final layer by independently changing the values of the pixels in each channel using different chaotic sequences generated from the three maps and the XORing operation. The efficiency of the proposed scheme is validated through key sensitivity, key space analysis, complexity analysis, entropy assessment, and tests, including horizontal, vertical, and diagonal correlation, MSE, PSNR, UACI, and NPCR. Moreover, experimental results and a thorough security analysis affirm that the proposed encryption technique has effectively attained confidentiality and robust resistance against various attacks.

## Introduction

Digital images are now indispensable for conveying information, due to their straightforwardness, ease of understanding, and suitability for facilitating communication across languages, ages, and geographical barriers. Large volumes of images are being transferred across various industries in significant quantities, and they find extensive applications across diverse fields, including image forensics, image reconstruction, and image steganography. Yet, the comprehensive utilization of images has also sparked particular concerns. Critical images may encounter risks such as privacy breaches, data tampering, and malicious destruction. Therefore, transforming images into unrecognizable formats is an effective strategy to safeguard image information during transmission, ensuring protection against unauthorized access by adversaries^1,2^.

Image security methods are crucial for protecting sensitive visual data in an increasingly digital world. These methods typically involve techniques such as watermarking^3,4^, data hiding^5,6^, and encryption^7,8^ to ensure confidentiality, integrity, and authenticity. Secure transmission and storage are ensured through image encryption, which involves converting a natural image into a noise-like or texture-like representation. Numerous image encryption schemes have been proposed, including bit-level permutation^9,10^, DNA coding^11,12^, S-box^13,14^, compressed sensing^15,16^, frequency transformation^17,18^, and chaos theory^19–22^. Chaos theory originated in the realms of mathematics and physics during the 1960s. Today, it assumes a crucial role in image security by leveraging its intrinsic traits like random behavior, uncertainty, and nonlinearity, thus furnishing robust cryptographic properties, which endows it with a distinctive advantage in image encryption.

Numerous image encryption techniques have been presented in the last thirty years. Unfortunately, from a contemporary cryptanalysis perspective, certain algorithms have been classified as insecure to differing extents. An effective technique for encrypting images must possess an extensive keyspace to withstand statistical attacks, accommodate images of varying sizes and types, and diminish the correlations in plaintext images. Moreover, several cryptanalysis investigations have been conducted to evaluate the effectiveness of image encryption and provide recommendations for improving its security, as indicated in^23,24^, which helps researchers enhance their cryptosystems.

Image encryption algorithms are classified into two main groups depending on the number of images encrypted: single-image encryption (SIE) and multiple-image encryption (MIE). As the number of captured and shared images continues to increase, there is a rising need for encryption algorithms that can effectively secure multiple images. MIE is a more robust encryption strategy developed to cope with the development of data, notably images used every day in most fields, and safeguard multiple images concurrently, rather than encrypting them one at a time as in SIE. This solution employs a unifying cryptographic mechanism to manage various images comprising diverse types (e.g., grayscale, RGB, hyperspectral, or multi-dimensional images). Accordingly, MIE offers unified protection, which minimizes the impact of breaches and increases complexity compared to isolated protection of single images and reduces computational costs compared to the processing required for each single image separately. The encryption ensures that all images are protected under a consistent security framework, enabling both efficiency and better security. MIE is designed to meet the special requirements of encrypting and securely transmitting multiple images. Examples of applications include remote sensing and satellite imaging, secure cloud storage and IoT devices, medical imaging, and surveillance systems.

Over recent years, there has been a noticeable increase in the publication of research focused on multiple image encryption algorithms. For example, Rasul et al.^25^ combined all images into a single large image before converting them into one-dimensional data for encryption. However, based on experimental findings, the security level achieved was considered unsatisfactory. Patro et al.^26^ proposed a new encryption method for multiple images using cross-coupling chaos. It employs an iterative process of crosscoupling a chaotic map system for merging images’ diffusion and replacement operations. The scheme proposed by Gao et al.^27^ encrypts multiple images optically; however, optical encryption entails high costs and poses practical challenges and limitations. Sahasrabuddhe et al.^28^ created a 3D cube by combining multiple plaintext images. They then rearranged this cube slice by slice, rotated it, and applied an XOR operation with a chaotic cube to generate the 3D ciphertext cube. Nevertheless, this algorithm necessitates using multiple images to form a 128 × 128 × 128 cube, thereby enforcing a predetermined quantity and dimension of images. Zhang et al.^29^ utilized DNA coding to introduce a multiple-image encryption algorithm, incorporating a chaotic Logistic map and Rossler system in their approach. This algorithm applies to grayscale images of uniform size.

Gao et al.^30^ introduce a fast encryption scheme for multiple images designed for grayscale and color images. Nevertheless, this scheme has a limitation of encrypting a maximum of 3 grayscale images simultaneously, rendering it unsuitable for encrypting more than three images. Laiphrakpam et al.^31^ introduced an enhanced chaotic map used for encrypting multiple images. This scheme combines various images into three planes. Then, an amplified sine map is used to generate a dynamic permutation table used to perform cyclic shift transformation, and a chaotic sequence is used to diffuse a permuted image by XORing it with this permuted image. However, this encryption scheme can only encrypt grayscale images. Sabir & Guleria^32^ designed a novel multiple-image encryption technique utilizing the Affine Hill cipher, reality-preserving two-dimensional discrete fractional Hartley transform, and a generalized two-dimensional Arnold map.

Xu^33^ proposed a multiple-image encryption algorithm based on orthogonal arrays with strength 3, in which the diffusion process is implemented by the pseudo-random sequences generated by the orthogonal array to modify the pixel contents of images. Then the permutation is designed to further scramble the image data based on orthogonal arrays (OAs) with strength 3. Zhang & Liu^34^ applied a multiple-image encryption algorithm using stereo Zigzag transformation by creating a cube from multiple grayscale images, then the Henon map is used to randomly select the starting point of the two-dimensional Zigzag transformation, which is used to scramble the cube; finally, a chaotic sequence is used to disperse the scrambled images to get the encrypted images randomly. While these methods demonstrate efficacy, some algorithms possess limited key spaces, making them susceptible to brute-force attacks. Additionally, optical methods prove costly, and some schemes fail to adequately conceal the statistical properties of images. Another critical concern is the limitation of algorithms in the number of images that can be encrypted simultaneously.

According to that, this paper proposes an MIE algorithm based on channel randomization and multiple chaotic maps. While the primary contributions of this study can be specified as follows:

1. The proposed algorithm can simultaneously encrypt a batch of color images, accommodating any number greater than 2. However, to fully benefit from the batch concept and the channel randomization process, the number of images should be at least 6.
2. The cipher images are closely interconnected within the same batch. If one or a portion of the cipher images is lost, the remaining cipher images within the same batch can assist in recovering partial information about the lost image.
3. A channel randomization process is proposed to improve randomness and break channel correlations.
4. A batch scramble process is proposed in each channel independently based on a unique key generated from a unique chaotic map. This helps increase the correlation divergence between channels and break the intra-channel correlation. Other different chaotic keys are used in the diffusion process to diffuse the batch channels independently, helping to hide the plain image features completely.

The proposed multiple-image encryption scheme addresses the security challenges highlighted by Li et al.^24^ by incorporating a batch concept with a channel randomization process and the chaotic behavior of three chaotic maps. These maps enhance system complexity, improving key sensitivity and keyspace. Additionally, integrating extra encryption layers further elevates the security level.

The rest of this paper is organized as follows: Sect. “Chaotic Maps” introduces the chaotic maps utilized. Section “The proposed MIE Algorithm” proposes a Multiple Image Encryption (MIE) algorithm. Section “Performance analysis” presents the experiment and algorithm analysis. Finally, Sect. “Conclusion” provides the conclusion of the paper.

## Chaotic maps

Chaos-based systems are generally suitable for creating image encryption methods because of their highly effective features, such as pseudorandom, unpredictability, and aperiodic behaviors^35,36^. Chaotic maps produce random sequences for the two fundamental encryption processes in the image encryption scheme: image permutation and diffusion^37^. The Baker system, 2-D logistics, and Henon maps are used in the suggested scheme.

### Baker map

The Baker map is one of the most commonly employed chaotic maps in image encryption^38^. Its equation for the 2-dimensional representation can be expressed as

1 $$\left( {{X_{n+1}},{Y_{n+1}}} \right)=~\left\{ {\begin{array}{l} {\left( {\frac{{{X_n}}}{\alpha },\alpha {Y_n}} \right),~0<X~ \le \alpha } \\ {\left( {\frac{{({X_n} - \alpha )}}{{\left( {1 - \alpha } \right)}},~\left( {1 - \alpha } \right){Y_n}+\left( {1 - \alpha } \right)} \right),~\alpha <x \le 1} \end{array}} \right.$$

In this equation, $\alpha$ denotes the system parameter, while ${X_n}\,{\text{and}}\,{Y_n}$ represent the state variables. The system exhibits chaotic behavior and can generate two chaotic sequences: {${x_n}$} and {${y_n}$}.

### Henon map

Due to its advantageous qualities, including the Lyapunov exponent, random behavior, and uniform non-variation of the density variable, the Henon map is highly recommended for cryptographic applications. Henon^39^, a French mathematician and astronomer, proposed a two-dimensional Henon map, which transforms a point (${{\text{U}}_{\text{n}}}$, ${{\text{T}}_{\text{n}}}$) into (${{\text{U}}_{{\text{n}}+1}}$, ${{\text{T}}_{{\text{n}}+1}}$) as

2 $$\begin{aligned}{U_{n+1}}&=1 - \mu U_{n}^{2}+{T_n}\\ {T_{n+1}}&=\rho {U_n}\end{aligned}$$

where $\mu$ and $\rho$ represent system parameters, ${U_n}$ and ${T_n}~$, means that the state values of the system at time n. When $\mu$ = 1.4 and $\rho$ = 0.3, the map is ensured to be chaotic^40^. The Henon chaotic map can provide confusion and flat histogram, is computationally efficient, and exhibits near optimal randomness properties.

### 2D logistic map

The two-dimensional logistic map^41,42^ boasts several cryptographic attributes, such as fewer periodic windows in bifurcation diagrams and a wider spectrum of parameters conducive to chaotic behaviors. Its widespread application stems from its high sensitivity to initial parameters, simple expression, rapid computational speed, and robust chaotic characteristics. The 2-D logistic map^43^ is defined as

3 $$\begin{aligned}{S_{n+1}}&=\left( {{i_1}{S_n}} \right)\left( {1 - {S_n}} \right)+\left( {{j_1}Z_{n}^{2}} \right)\\ {Z_{n+1}}&=\left( {{i_2}{Z_n}} \right)\left( {1 - {G_n}} \right)+{j_2}\left( {S_{n}^{2}+{S_n}{Z_n}} \right)\end{aligned}$$

Where 2.75 <${i_1}$ ≤ 3.4, 2.75 < ${i_2}$ ≤ 3.45, 0.15 < ${j_1}$ ≤ 0.21, 0.13 < ${j_2}$ ≤ 0.15, ${S_n}$, ${Z_n}$ ∈ (0, 1]. The control parameters ${i_1}$, ${j_1}$, ${i_2}$, ${j_2}$ and the initial values ${S_0}$, ${Z_0}$, can be used to generate random secret keys for encrypting images, which raise the keyspace level.

## The proposed MIE algorithm

### Encryption process

**Fig. 1:**
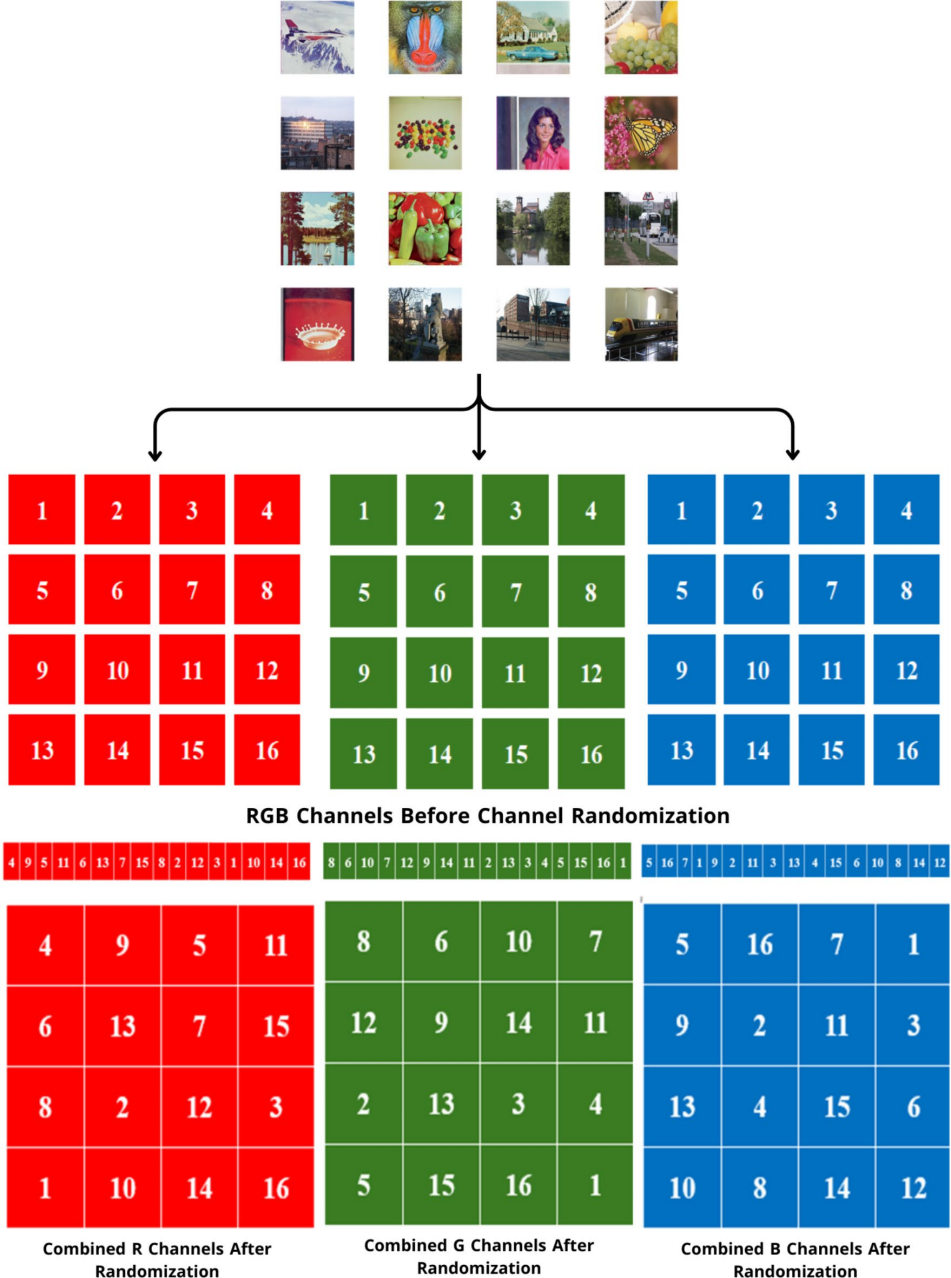
Channel randomization.

Three channels of the uni-size, $M \times N$, were created by decomposing the input color images (I_1_,…, I_n_) of size, $M \times N \times 3$. The images in each channel are then arranged randomly before being combined to form a big (batch) image according to *Algorithm 1*, which represents layer1 of the encryption process. Figure 1 shows a visualized flowchart for the Randomization process.


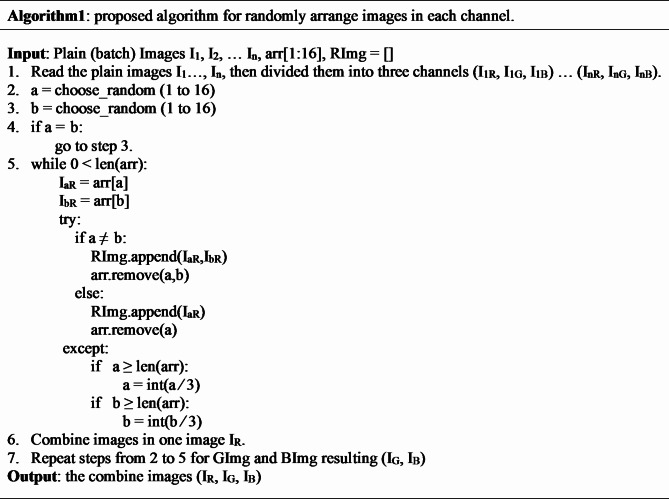


**Fig. 2:**
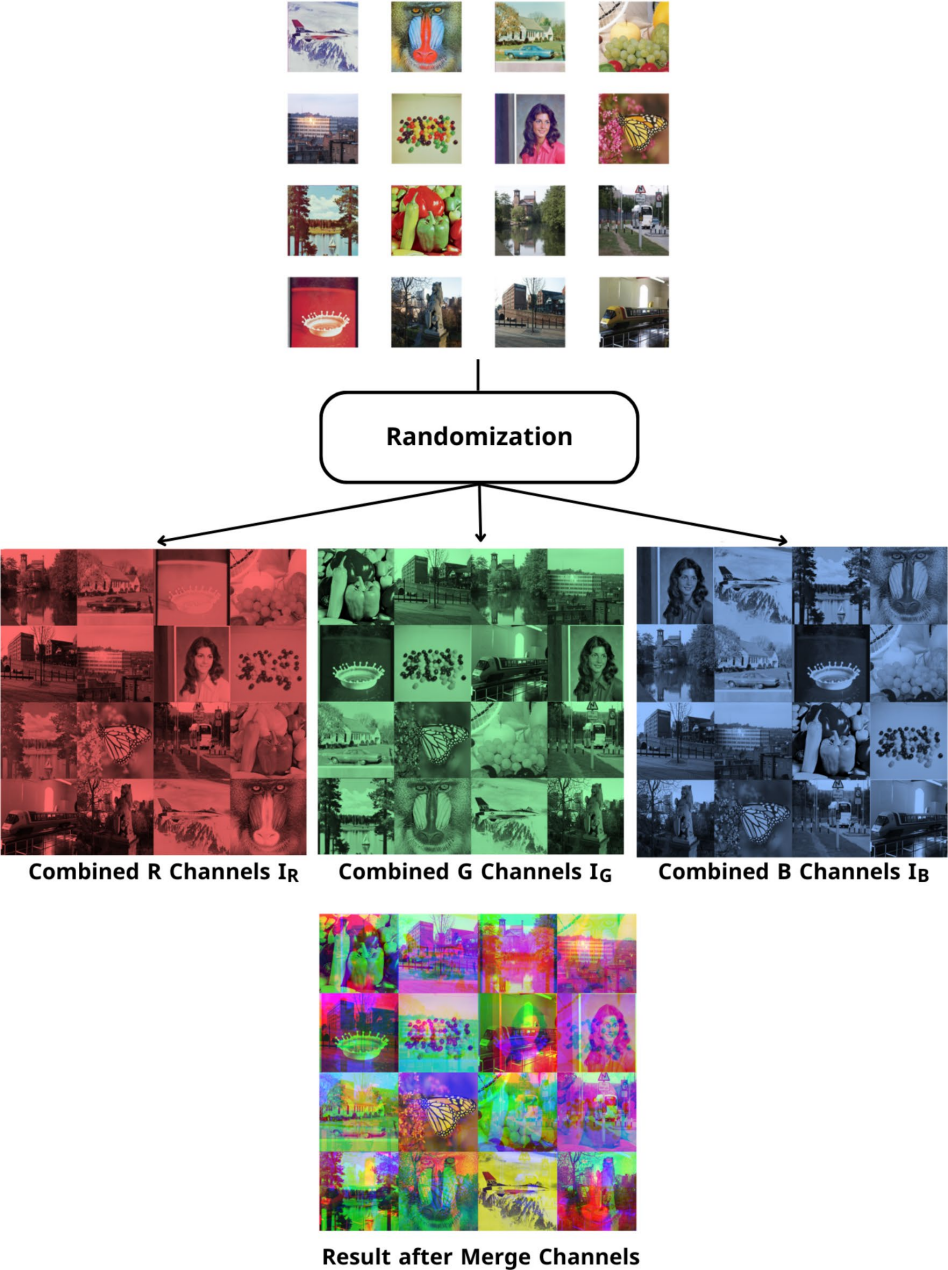
Example for channel randomization.

If combined channels $\left( {{I_R},{I_G},{I_B}} \right)$ are merged, we get a completely different plain augmented image, as shown in Fig. 2, which is an entrance to the second layer. In the second layer, the scramble process is performed. Image scrambling refers to altering the position of pixels in an image without changing their values. In our algorithm, the images $\left( {{I_R},{I_G},{I_B}} \right)$ are scrambled independently by the sequences $\left( {x,u,s} \right)$ generated by Baker, Henon, and 2D Logistic chaotic maps as following steps:

I. Reading plain gray images $\left( {{I_R},~{I_G},~{I_B}} \right)$of size $\left( {M \times N} \right),$ then convert them to three vectors: ${V_R}$, ${V_G}$, and ${V_B}$.
II. Key Generation:
  1. Let $m= M\times N$, and obtain N_0_ using the following equation
    4 $${N}_{0}=\frac{1}{m+{2}^{23}}\sum_{j=0}^{m}{(V}_{Rj}+{V}_{Gj}+{V}_{Bj})+(m)$$
  2. Generate the initial parameters
    5 $$\begin{aligned}{x}_{0}&=\frac{{e}^{{N}_{0}\:mod\:2}}{10}\\{u}_{0}&=\frac{{e}^{{N}_{0}\:mod\:2}}{10}\\{s}_{0}&=\frac{{e}^{{N}_{0}\:mod\:2}}{10}+3.999\end{aligned}$$
  3. Input the secret (encryption) key $\alpha,\mu,\rho,{i}_{1},{j}_{1},{i}_{2},{j}_{2}$ and ${x}_{0},{u}_{0},{s}_{0}$ into the mentioned chaotic maps. Iterate them $m$ times using (1), (2), and (3) system equations to obtain chaotic sequences $x,y$, $u,t,s,z$.
  4. Separate the chaotic sequences into two groups, ${S}_{k}(x,u,s)$for the permutation process and ${D}_{k}(y,t,z)$ for the diffusion process.
III. Decimal Permutation:
  In this stage, the vector channels $\:{V}_{R}$, $\:{V}_{G}\,{\text{and}}\,{V}_{B}$ are shuffled using the sequences $\:x,u,s$ in the following way:
  1. Initializing an empty matrix $PI$ of size $1\times n$ to store the permuted image.
  2. For each pixel in ${V}_{R}$, swap its position according to ${S}_{k1}$ and update the corresponding pixel in $PI$.
    6 $${PI}_{R}\left[l\right]\leftarrow {V}_{R}\left[{S}_{k1}\left(l\right)\right]\quad where \quad l\in [0,m]$$
  3. Reshape ${PI}_{R}$to size ($M\times N$), resulting in the permuted red channel ${I}_{R}$.
  4. Repeat steps 2 and 3 for ${V}_{G}\,and\,{V}_{B}$, following these operations:
    7 $$\left\{\begin{array}{l}{PI}_{G}\left[l\right]\leftarrow {V}_{G}\left[{S}_{k2}\left(l\right)\right]\quad where\quad l\in [0,m]\\ {PI}_{B}\left[l\right]\leftarrow {V}_{B}\left[{S}_{k3}\left(l\right)\right]\quad where\quad l\in [0,m]\end{array}\right.$$
    This form emphasizes that the green and blue channels, $\:{PI}_{G}$ and $\:{PI}_{B}$, are updated by swapping the positions defined by $\:{S}_{k2}\left[l\right]$ and $\:{S}_{k3}\left[l\right]$, respectively.
  5. Finally, reshape ${PI}_{G},{PI}_{B}$to size ($M\times N$), producing the permuted green and blue channel ${I}_{G}\,and\,{I}_{B}.$

**Fig. 3:**
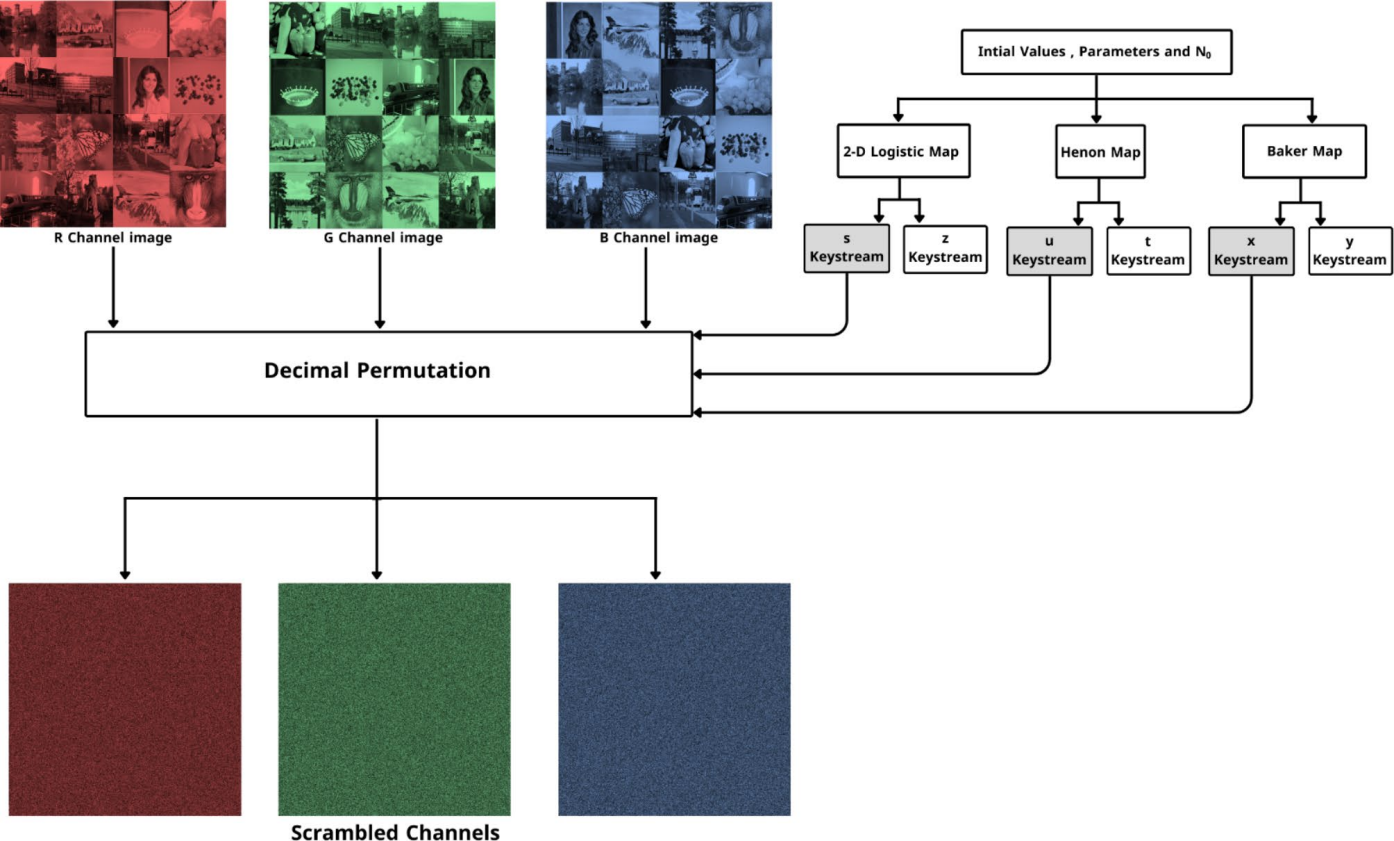
Pixels permutation process.

**Fig. 4:**
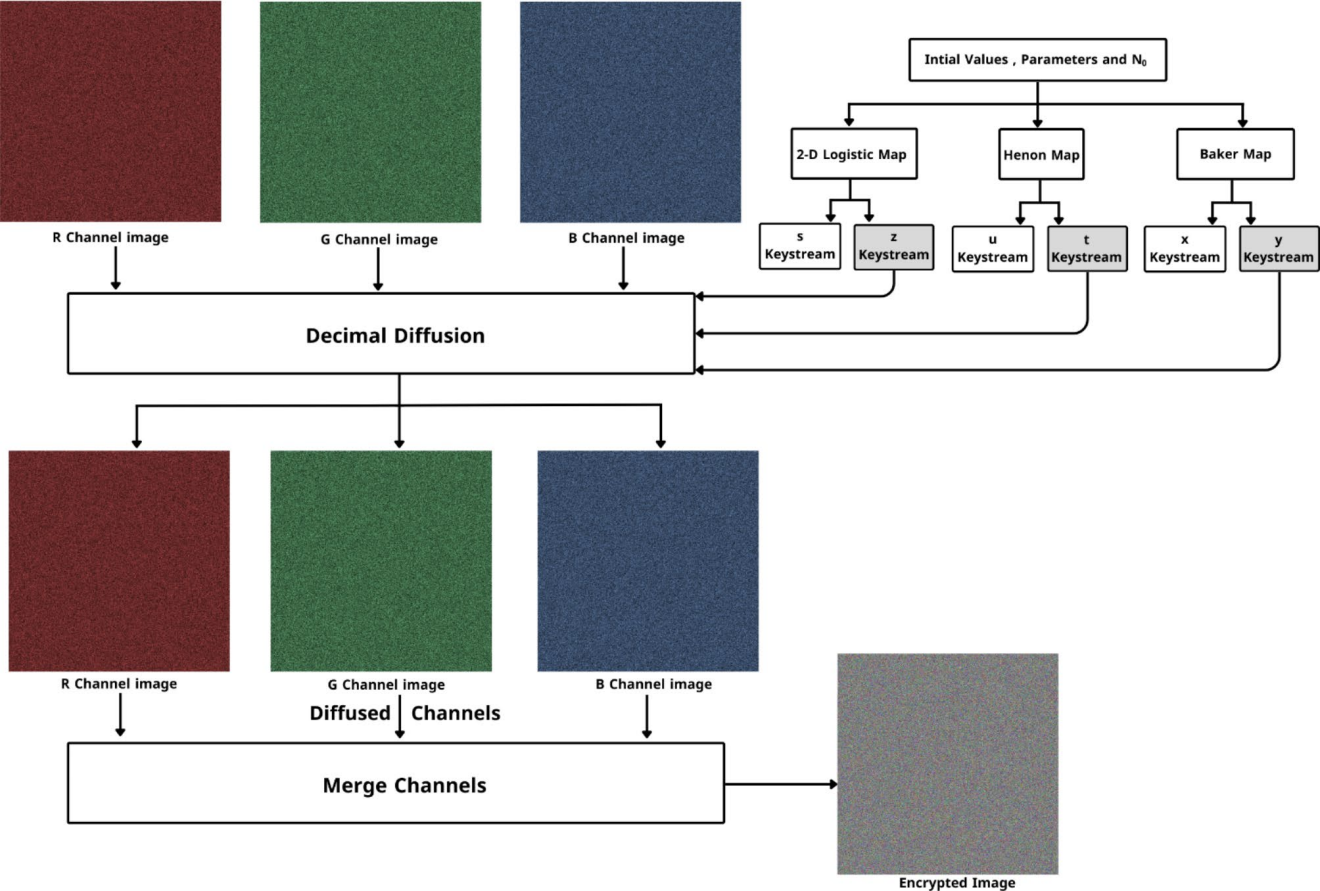
Pixels diffusion process.

**Fig. 5:**
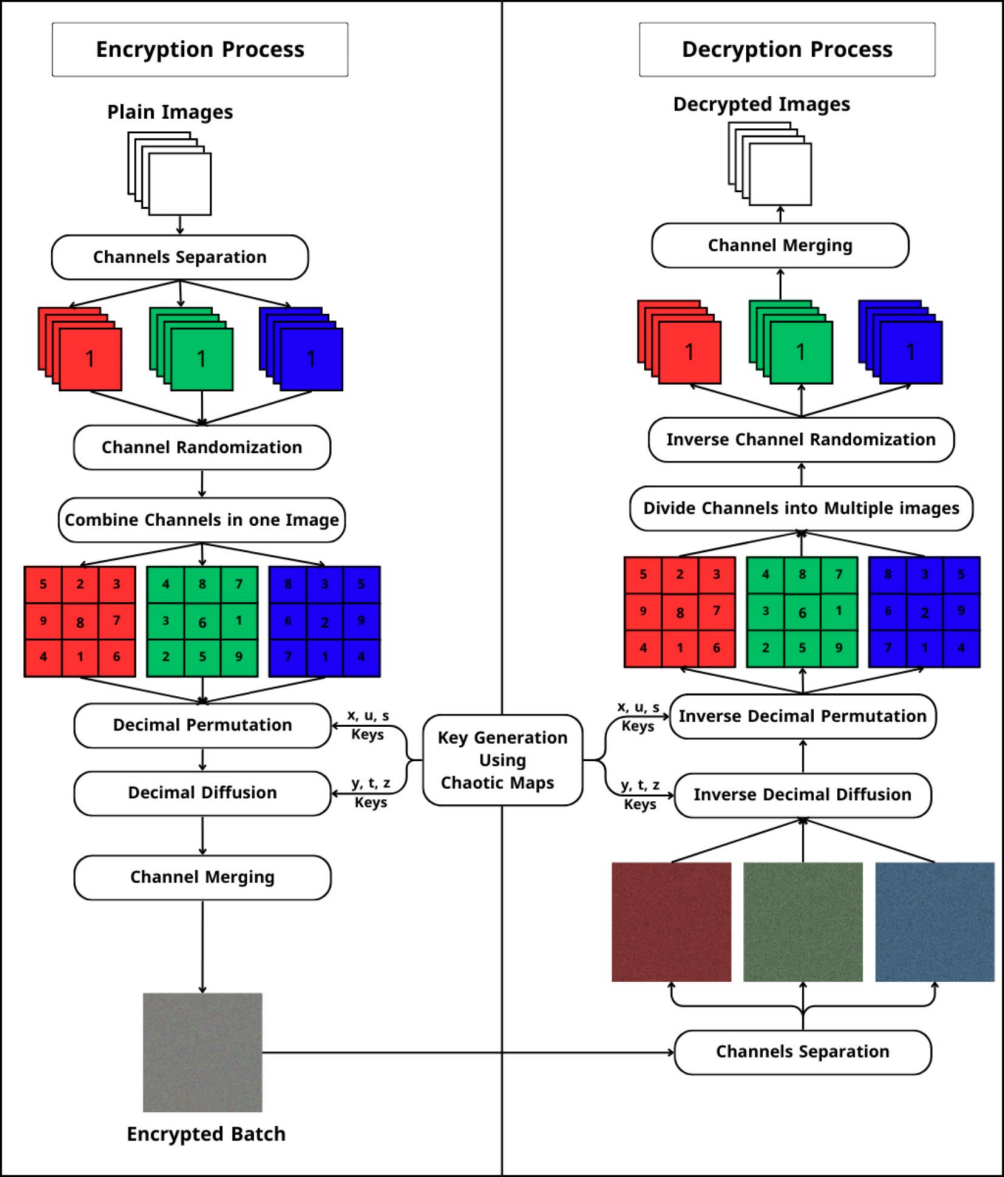
Full MIE encryption and decryption process.

After this step, which represents the final step in layer two $\:{{I}_{R},I}_{G},\:{I}_{B}$, channels served as an entrance to the third layer. The second layer scheme is shown in Fig. 3. The third layer is where the diffusion process occurs. The third layer scheme is shown in Fig. 4. Throughout the diffusion process, the original values of (I_R_, I_G_, and I_B_) permuted channels pixels are diffused independently by the sequences ($\:y,\:t,\:z$) generated by Baker, Henon, and 2D Logistic chaotic maps in the following step:

IV. Decimal Diffusion:
  In this process, the pixel values of the permuted channels $\:\:{I}_{R}$, $\:{I}_{G}$, and $\:{I}_{B}$, are modified using a chaotic matrix:
  1. Initialize an empty matrix $\:PD$ with dimensions $\:M\times\:N\times\:3$ to represent the diffused channels.
  2. For each pixel in ${I}_{R}$, perform a bitwise XOR operation with the chaotic matrix ${D}_{k1}$ resulting in the red diffused channel ${PD}_{R}$.
    8 $${PD}_{R}\left[l\right]={I}_{R}\left[l\right]\oplus {D}_{k1}\left[l\right]\quad where\quad l\in [0,M\times N]$$
  3. Repeat the XOR operation for the green and blue channels, ${I}_{G},\,{I}_{B}$ using chaotic matrices ${D}_{k2}\,and\,{D}_{k3}$, respectively:
    9 $$\left\{\begin{array}{l}{PD}_{G}\left[l\right]={I}_{G}\left[l\right]\oplus {D}_{k2}\left[l\right]\quad where\quad l\in \left[0,M\times N\right]\\ {PD}_{B}\left[l\right]={I}_{B}\left[l\right]\oplus {D}_{k3}\left[l\right]\quad where\quad l\in \left[0,M\times N\right]\end{array}\right.$$
V. Finally, merge the diffused ${PD}_{R},\,{PD}_{G,}$ and ${PD}_{B}$ channels into the matrix $PD$, forming the fully encrypted batch image. The complete MIE encryption process is shown in Fig. 5.

### Decryption process

Decryption is the process that reverses encryption, restoring encrypted data to its original, unencrypted form. This procedure will enable the recovery of the input image from the encrypted one. The following steps can be used to summarize the decryption process:

1. Split the encrypted image into three $\:{I}_{R}$, $\:{I}_{G}\,{\text{and}}\,{I}_{B}$ channels.
2. Dediffused $\:{I}_{R}$, $\:{I}_{G}$, and $\:{I}_{B}$ channels by XORing them with $\:{D}_{k}$ sequences to get scrambled $\:{V}_{R}$, $\:{V}_{G}$, and ${V}_{B},$ channels.
3. Descramble $\:{V}_{R}$, $\:{V}_{G}\,{\text{and}}\,{V}_{B}$ channels by retrieving the original positions of each pixel using $\:{S}_{k}$ sequences to get the original plain channels $\:{I}_{R}$, $\:{I}_{G}$, and $\:{I}_{B}$.
4. Divide each channel $\:{I}_{R}$, $\:{I}_{G}$, and $\:{I}_{B}$, to $\:n$ images.
  $$\begin{aligned}\left\{\begin{array}{l}{I}_{Rq},{I}_{Rp},\:\dots\:\dots\:\dots\:,{I}_{Re}\\{I}_{Ga},{I}_{Gb},\:\dots\:\dots\:\dots\:,{I}_{Gc}\\{I}_{Bf},{I}_{Bd},\:\dots\:\dots\:\dots\:,{I}_{Bo}\end{array}\right.\\ where\:q,p,e,a,b,c,f,d,o \in [0,n]\end{aligned}$$
5. Rearrange $\:n$ images to get the original order of images in each channel.
  $$\left\{\begin{array}{l}{I}_{R1},{I}_{R2},\:\dots\:\dots\:\dots\:,{I}_{Rn}\\{I}_{G1},{I}_{G2},\:\dots\:\dots\:\dots\:,{I}_{Gn}\\{I}_{B1},{I}_{B2},\:\dots\:\dots\:\dots\:,{I}_{Bn}\end{array}\right.$$
6. Merge all three channels of the same image to obtain the original image (this step is done $\:n$ times with the same number of images). The full MIE decryption process is shown in Fig. 5.
  $$\begin{array}{c} {merge\left( {{I_{R1}},{I_{G1}},{I_{B1}}} \right)} \\ {merge\left( {{I_{R2}},{I_{G2}},{I_{B2}}} \right)} \\ \cdot \\ \cdot \\ \cdot \\ {merge\left( {{I_{Rn}},{I_{Gn}},{I_{Bn}}} \right)} \end{array}$$

## Performance analysis

This section covers the results that demonstrate the suggested algorithm’s effectiveness. The experiments use a batch of 16 images with the size of ($256 \times 256$), which produces Mix_16 image of size ($1024 \times 1024$). Table 1 displays the test plain image utilized in the experiments.

**Table 1:** Batch of color images.

Name	Plain images	Batch image after layer 1	Size
Mix_16			$\:1024\times\:1024$

### Information entropy

An important analysis assesses the randomized and unpredictable nature of the proposed scheme. For every RGB color image channel, the information entropy should ideally be 8. The following formula can be used to estimate entropy:

10 $$H\left(X\right)=-\sum_{i=0}^{m}P\left(Xi\right)\,{{log}}_{2}(P\left(Xi\right))$$

Where *H*(*X*), represents the entropy value of the image $X$, P($X$_*i*_) denotes the probability of the symbol $X$_i_ appearing. An overview of the global and local entropy values derived from the batch image analysis is provided in Table 2. Local entropy computation was conducted using the approach described in^44^, utilizing 31 non-overlapping blocks of pixels. Each block contained 1936 pixels extracted from the encrypted image for local entropy evaluation. Since all local entropies fall within the acceptance intervals at significance levels of 5%, 1%, and 0.1%, it can be inferred that the proposed encryption algorithm demonstrates high resilience against entropy attacks. Table 3 displays various experimental entropy results of the MIE using our scheme and recent algorithms.

**Table 2:** Information entropy for colored-batch-image.

Image	Global entropy			Local entropy		
	*R*	G	B	*R*	G	B
Mix_16	7.9999	7.9999	7.9998	7.9033	7.9020	7.9032

**Table 3:** Comparison of different MIE algorithms.

Image	Entropy	NPCR (%)	UACI (%)	PSNR	MSE
Proposed	7.9999	99.6255	33.5281	8.1506	9864.3
Ref^25^	7.9994	99.6653	33.3857	−	−
Ref^26^	7.9993	99.6167	33.4772	9.4339	7411.7
Ref^45^	7.9998	99.6060	33.5126	8.8260	8522
Ref^29^	7.9994	99.64	33.38	−	−
Ref^30^	7.9993	99.6142	33.4656	−	−
Ref^31^	7.9998	99.62	33.42	8.3862	−
Ref^33^	7.9993	99.6127	33.4765	9.1070	7987
Ref^34^	7.9998	99.5908	33.4210	−	−
Ref^46^	−	99.61	33.39	−	−

### Correlation analysis

The association between diagonal, horizontal, and vertical pixels should be eliminated using an effective encryption technique. For estimating the correlation $\:{r}_{i,j}$ of 10,000 randomly selected adjacent pixel pairs $\:(i,j)$ from the cipher image, the following equations were utilized:

11 $$\:{r}_{i,j}=\frac{Co\left(\right(i-Co\left(i\right)\left(j-Co\left(j\right)\right))}{\sqrt{D\left(i\right)D\left(j\right)}}$$

where

12 $$\:Co\left(i\right)=\frac{1}{N}\sum\:_{n=1}^{N}{i}_{n},D\left(i\right)=\frac{1}{N}\sum\:_{n=1}^{N}{{(i}_{n}-Co\left(i\right))}^{2}$$

**Fig. 6:**
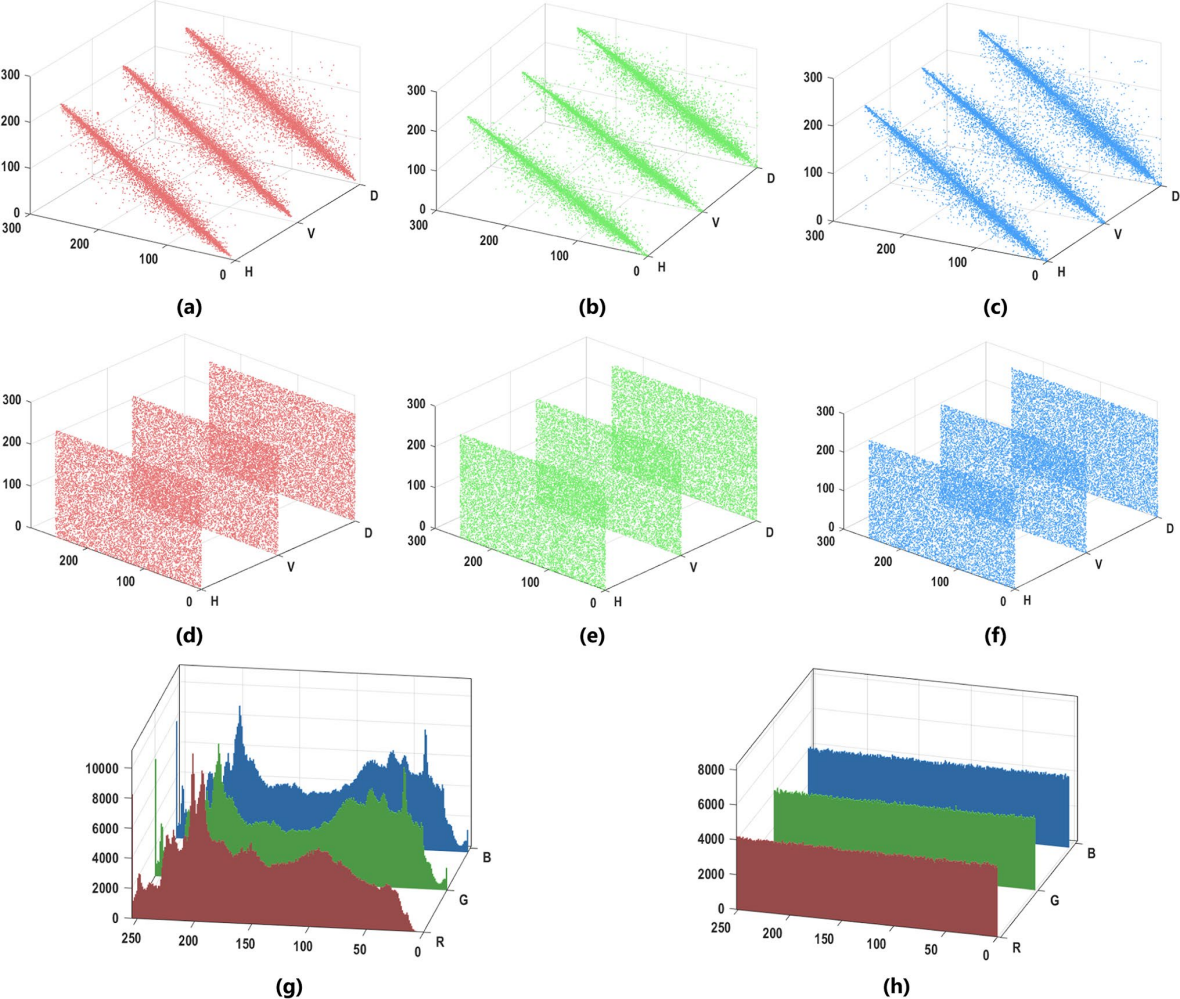
The correlation of plain Mix_16 batch image (**a**, **b**, **c**) and corresponding cipher image (**d**, **e**, **f**). Histogram Analysis of plain Mix_16 batch image and corresponding ciphered image (**g**, **h**).

Figure 6 displays the pixel distributions along the three orientations of the (RGB) plain and encrypted image. Table 4 identifies the plain and cipher images’ neighboring pixel values in three different orientations. The results are approaching 0, exhibiting that the pixels are highly dissimilar. Table 5 displays various experimental correlation results of the MIE using our scheme and recent algorithms.

**Table 4:** Correlation of plain and cipher batch images.

Image	Direction	Plain Image			Cipher Image		
		R	G	B	R	G	B
Mix_16	H	0.9690	0.9742	0.9704	0.0006	-0.0094	0.0009
	V	0.9663	0.9703	0.9720	0.0002	-0.0009	0.0003
	D	0.9463	0.9519	0.9518	0.0012	-0.0014	-0.0012

**Table 5:** Comparison of correlation for different MIE algorithms.

	Proposed	Ref^25^	Ref^26^	Ref^45^	Ref^29^	Ref^30^	Ref^31^	Ref^33^	Ref^34^	Ref^46^
Direction										
H	0.0005	0.0009	– 0.0004	− 0.0003	−0.0029	0.0021	0.0024	0.0129	-0.0007	− 0.0019
V	-0.0001	0.0015	– 0.0019	0.0011	−0.0049	0.0029	0.0055	0.0038	0.0002	− 0.0018
D	-0.0004	0.0008	0. 0015	0.0013	−0.0021	0.0023	-0.002	0.0011	0.0010	− 0.0121

### Histogram analysis

An image’s histogram visually represents the distribution of grayscale values according to their frequencies. The histogram should be flat, and the distribution of the cipher images should be uniform. Figure 6 illustrates the histograms for the (Mix_16) batch image and its corresponding cipher image.

Although visually examining image histograms helps assess the robustness of an encryption system against statistical attacks, quantitative analysis offers more comprehensive and detailed insights. The Chi-square (*χ*2) goodness-of-fit statistic is commonly employed to assess the uniformity of image histogram, calculated as follows:

13 $${{\upchi}}^{2}=\sum_{l=0}^{L-1}\frac{F\left(l\right)-E\left(l\right)}{E\left(l\right)}$$

where

14 $$E\left(l\right)=\frac{W\times H}{L}\,\, \forall\, l$$

Let $\:L$, denote the number of levels in an 8-bit channel (256 levels); $\:F\left(l\right),\:$represents the observed frequency of each level $\:l$, while $\:E\left(l\right)$ denotes the expected frequency for perfectly uniform distribution in an image of size $\:W\times\:H$. To pass the chi-square test, $\:{{\upchi\:}}^{2}$ must be smaller than $\:{{\upchi\:}}_{{\upalpha\:}}^{2}\left(df\right)$, where $\:{{\upchi\:}}_{{\upalpha\:}}^{2}\left(df\right)$, is the critical chi-square value, is the significance level, and $\:df$is the degree of freedom of the chi-square distribution. Using $\:\alpha\:=0.05\:$ and $\:df=L-1=255$, the critical chi-square value is ${{\upchi}}_{0.05}^{2}\left(255\right)=293.247$^47^. Table 6 displays the ${{\upchi}}^{2}$ results for the ciphered image. The effective interval of Chi-square is [210.7918, 293.2478]^48^. Hence, ${{\upchi}}^{2}$ values smaller than 293.247 demonstrate that the encrypted image’s histogram is quite similar to a uniform histogram and is robust to histogram analysis.

**Table 6:** Findings from the chi-square test.

Image	Plain-Image			Cipher-Image			Conclusion
	R	G	B	R	G	B	
Mix_16	214080	173110	173110	249.375	258.023	237.962	Pass

### Mean square error (MSE) and peak signal-to-noise ratio (PSNR)

For a cipher image to be considered effective, it should exhibit significant deviation from its original form. The Mean Square Error (MSE) measures the total squared discrepancy between the original and corresponding cipher image, computed as follows:

15 $${r}_{i,j}=\frac{1}{W\times H}\sum_{i=0}^{W}\sum_{j=0}^{H}{(P\left(i,j\right)-E\left(i,j\right))}^{2}$$

Where $P\left(i,j\right),$ is the value of the pixels of the plain image, and $E(i,j)$ is the encrypted pixel value at position $(i,j)$ in the cipher image. The MSE value can be used as a criterion to determine the encryption level of a cryptosystem. The encryption security increases with a larger MSE scale. PSNR analysis determines the quality level of encryption. A larger scale suggests that the encrypted image closely mirrors the original image. Therefore, a smaller PSNR value indicates more robust encryption for a cryptosystem. It can be described as follows:

16 $$PSNR=20\times\,{\text{log}}_{10}[255/\sqrt{MSE}]$$

The MSE and PSNR values in Table 7 illustrate the challenge of extracting the plain image from the cipher image without determining the secret key. Table 3 displays various experimental MSE and PSNR results of the MIE using our scheme and recent algorithms.

**Table 7:** The mean squared error (MSE) and peak signal-to-noise ratio (PSNR) values for the encryption process.

Image	MSE			PSNR		
	*R*	G	B	*R*	G	B
Mix_16	9624	9987	9982	8.2974	8.1363	8.1385

### Data loss

**Fig. 7:**
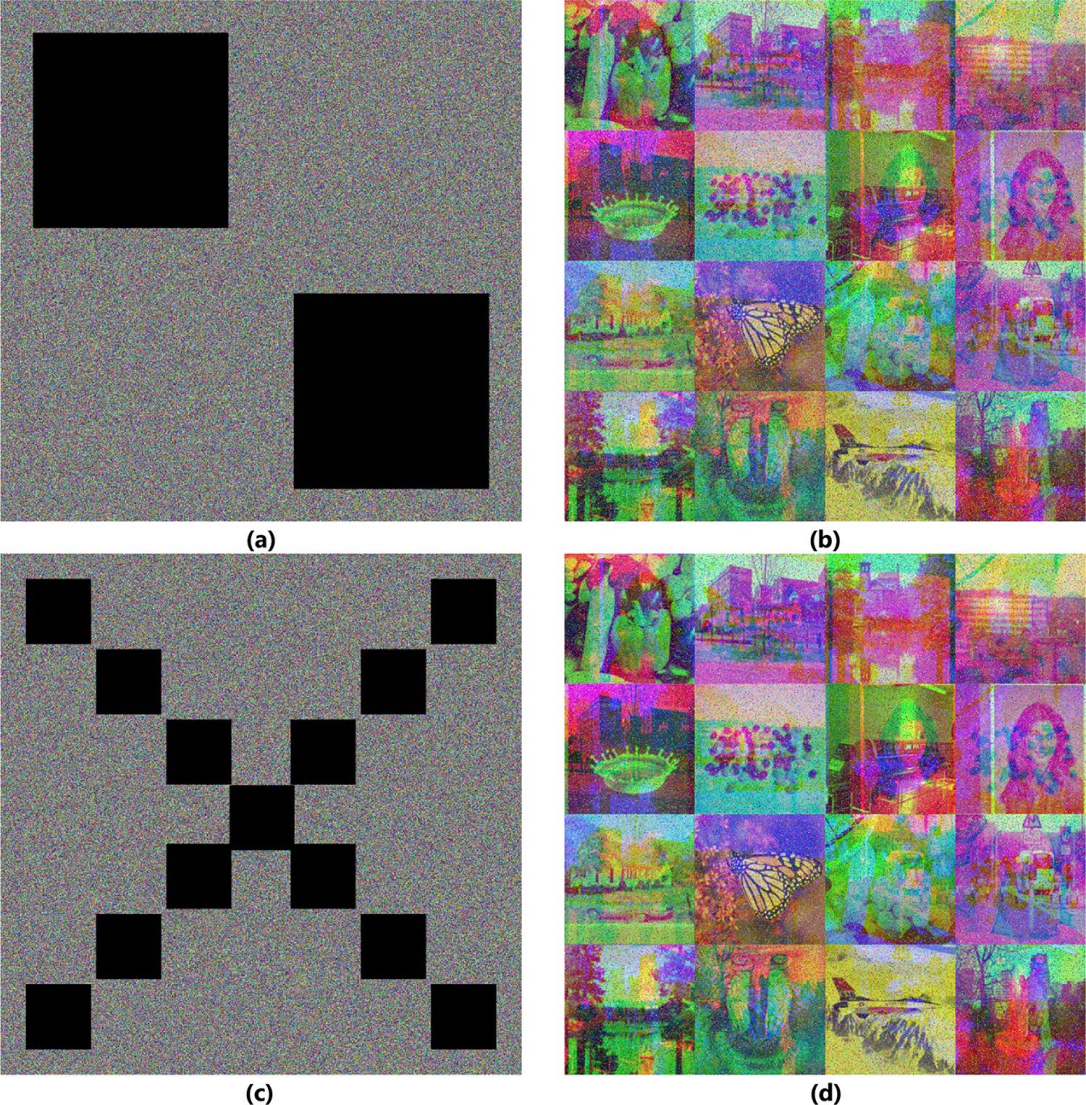
Data loss. Damaged cipher Mix_16 image with the cut of two boxes of size $\:384\times\:384$ and corresponding decrypted image (**a**, **b**), and with the cut of 13 boxes of size $\:128\times\:128\:$and corresponding decrypted image (**c**, **d**).

Images lose data when they are transmitted or stored. Part of the data is lost in cipher images, which are subsequently decrypted using the decryption process. Mix_16 (1024 × 1024) is the test case used in the experiment. The results of experiments partially destroying cipher image and then decrypting this batch image are displayed in Fig. 7. The findings indicate that the decrypted images retain their most authentic features, implying that the proposed technique effectively withstands this attack.

### Noise attack

**Fig. 8:**
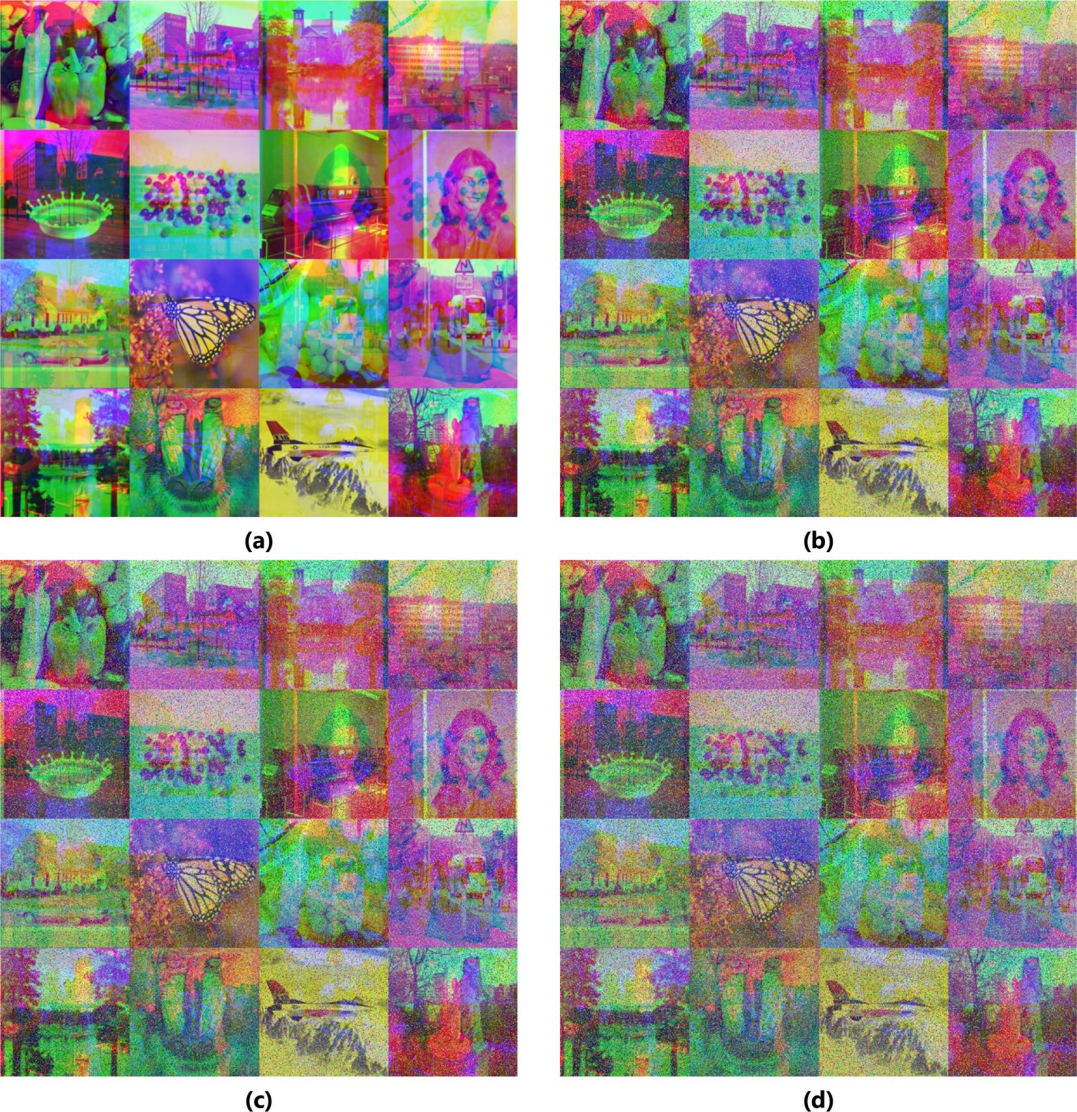
Noisy attack. (**a**) Mix_16 batch plain-image (**b**) after adding 0.2 (**c**) after adding 0.3 (**d**) after adding 0.4 Salt and Pepper noise to cipher image.

In addition to losing data, noise attacks images when transmitted or stored. To implement the noise attack idea, add noise to the cipher-batch image in different intensities—salt and pepper—and then decrypt this batch image. Mix_16 ($1024\times1024$) image is taken as a test case in this experiment. Figure 8 shows the result of the experiment implementation. The results demonstrate that the image has the most authentic features after decryption, indicating that the suggested technique can withstand this attack.

### Differential attack

Within this paper, there is a deliberate utilization of two commonly acknowledged sensitivity measures, namely the Unified Average Changing Intensity (UACI) and the Number of Pixels Change Rate (NPCR), serving as pivotal tools for assessing the robustness against the differential attack. These measures are crucial in comprehensively gauging the system’s resilience to potential vulnerabilities. The definitions of UACI and NPCR are as follows:

17 $$\:\left\{\begin{array}{l}UACI=\frac{1}{w\times\:h}\times\:\sum_{x=0}^{w}\sum_{y=0}^{h}\frac{|{C}_{1}\left(x,y\right)-{C}_{2}\left(x,y\right)|}{255}\times 100\%\\ NPCR=\frac{1}{w\times h}\times \sum_{x=0}^{w}\sum_{y=0}^{h}D(x,y)\times 100\%\end{array}\right.$$

where

18 $$D\left(x,y\right)=\left\{\begin{array}{ll}0, &\quad {C}_{1}\left(x,y\right)={C}_{2}\left(x,y\right)\\ 1,& \quad{C}_{1}\left(x,y\right)\ne {C}_{2}\left(x,y\right)\end{array}\right.$$

The $\,{C}_{1\:}and\:\,{C}_{2\:}$are two cipher images whose corresponding plain image has one random pixel variance, and (*w*,*h*) represents the number of rows and columns. For the batch image (Mix_16), a single pixel is randomly selected, and its value is incremented by one before encryption. Table 8 shows the calculated values of UACI and NPCR for the batch image. The ideal values of NPCR and UACI are 99.6094070% and 33.4635070%^48^. In that case, it can be inferred that the encryption method is strong, as even a minor alteration in the algorithm’s input yields a markedly different output^49^. The outcomes suggest that our approach demonstrates robustness against differential attacks. Table 3 displays various experimental UACI and NPCR results of the MIE using our scheme and recent algorithms.

**Table 8:** UACI and NPCR of different batch images.

Image	UACI%			NPCR%		
	*R*	G	B	*R*	G	B
Mix_16	33.5122	33.4996	33.5723	99.6169	99.6265	99.6319

### Complexity analysis

Moreover, it’s worth noting that when evaluating the efficacy of encryption, considerations extend beyond just security testing alone. Indeed, delving into the complexity of an encryption algorithm emerges as a pivotal indicator^50^. The randomization and combined processes result in a time complexity of (3∗T), where T is the number of images in the batch. For *M* × *N* RGB image after the combine process, each chaotic map generates M∗N chaotic sequences, resulting in a time complexity of (3∗*M*∗*N*). The permutation process happens as pixel values from each channel of the original image are mixed, operating in (*M*∗*N*) time complexity. The resultant chaotic maps are XORed with the RGB channels of the confused channels. This process has the complexity of (*M*∗*N*). In summary, the computational complexity of our suggested technique is (3*T+3∗*M*∗*N*+*M*∗*N*+*M*∗*N*), which simplifies to (T+*M*∗*N*).

### Key space

For an image cryptosystem to be considered valid, it necessitates a key space of significant magnitude, ensuring that brute-force attacks are rendered impractical and unfeasible. The total number of different keys that can be used in an encryption scheme is known as the key space. In this paper, the secret key mainly consists of the initial values ($S,Z,U,T,X,Y$) and the parameters $(\alpha,\mu,\rho,{i}_{1},{j}_{1},{i}_{2},{j}_{2})$. Suppose the control parameters’ precision and initial values are 10^−15^. Therefore, the corresponding key space value is approx. ${({10}^{15})}^{13}>{2}^{100}$. Hence, the scheme possesses a sufficiently large key space to thwart brute force attacks effectively.

### Key sensitivity

**Fig. 9:**
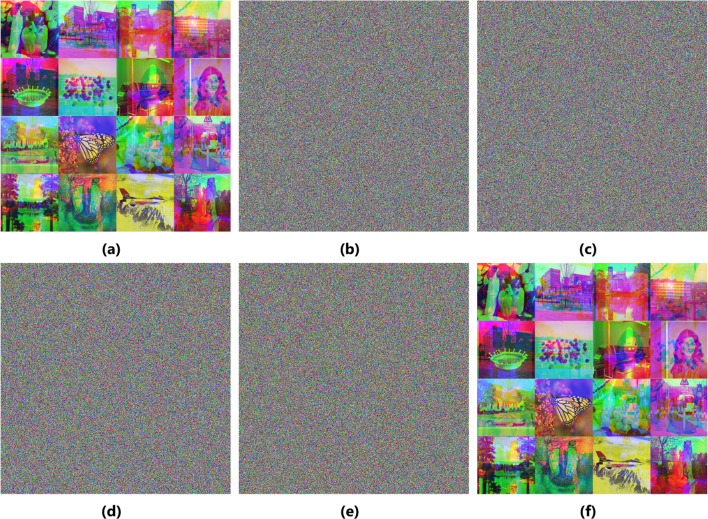
Key Sensitivity. (**a**) Plain Batch-image. (**b**) Cipher Image using K1. (**c**) Cipher Image using K2. (**d**) Difference between (**b**) and (**c**). (**e**) Decrypted Image (**b**) using the modified key K2. (**f**) Decrypted Image (**b**) using the original key K1.

The secret key should be completely different when generated using different initial conditions, especially when the difference between these initial conditions is minimal^51^. To carry out the key sensitivity analysis, key K1 is generated with the initial conditions to evaluate the proposed scheme’s key sensitivity. $\:{X}_{0},\:{U}_{0},\:{S}_{0}$ then, we change the initial conditions to $\:{X}_{0}+{10}^{-16}$, $\:{U}_{0}+{10}^{-16}$, $\:{S}_{0}+{10}^{-16}$ and then generate K2. Figure 9 illustrates the key sensitivity analysis for both keys.

## Conclusion

An effective batch color-image encryption method based on three layers and a combination of Baker, Henon, and 2D Logistic maps to encrypt colored batch images is proposed in this paper. All multiple images are separated into an RGB format in the first layer. After that, the images in each channel go through a randomization process to randomly ordered images before being combined to create a large image, or batch, which serves as the input for the layer that occurs after. In the second layer, Baker, Henon, and 2-D Logistic chaotic maps form sequences for randomly permuting pixels independently in each channel, producing a confusing image. In the last layer, the diffusion process is implemented by independently varying the values of the pixels in every channel using XORing operations and various chaotic sequences produced from the same maps. The outcomes of experiments that were put into practice provided insight into the effectiveness of the suggested encryption technique. They demonstrated that our system is highly resilient to attacks, including noise and data cuts attacks, has a sufficiently big keyspace to fend off brute force attacks, and has heightened sensitivity to both the plain image and keys. The encrypted image has an entropy near to perfect, a convenient histogram, and a low correlation. Thus, our suggested approach satisfies the encryption requirements.

## Data Availability

Data will be available upon request from the corresponding author.
